# Autophagy Takes Center Stage as a Possible Cancer Hallmark

**DOI:** 10.3389/fonc.2020.586069

**Published:** 2020-10-22

**Authors:** Jose G. Alvarez-Meythaler, Yoelsis Garcia-Mayea, Cristina Mir, Hiroshi Kondoh, Matilde E. LLeonart

**Affiliations:** ^1^Biomedical Research in Cancer Stem Cells Laboratory, Vall d'Hebron Research Institute (VHIR), Barcelona, Spain; ^2^Geriatric Unit, Graduate School of Medicine, Kyoto University, Kyoto, Japan; ^3^Spanish Biomedical Research Network Center in Oncology, CIBERONC, Barcelona, Spain

**Keywords:** autophagy, cancer, therapy, resistance, protective autophagy

## Abstract

Cancer remains one of the leading causes of death worldwide, despite significant advances in cancer research and improvements in anticancer therapies. One of the major obstacles to curing cancer is the difficulty of achieving the complete annihilation of resistant cancer cells. The resistance of cancer cells may not only be due to intrinsic factors or factors acquired during the evolution of the tumor but may also be caused by chemotherapeutic treatment failure. Conversely, autophagy is a conserved cellular process in which intracellular components, such as damaged organelles, aggregated or misfolded proteins and macromolecules, are degraded or recycled to maintain cellular homeostasis. Importantly, autophagy is an essential mechanism that plays a key role in tumor initiation and progression. Depending on the cellular context and microenvironmental conditions, autophagy acts as a double-edged sword, playing a role in inducing apoptosis or promoting cell survival. In this review, we propose several scenarios in which autophagy could contribute to cell survival or cell death. Moreover, a special focus on novel promising targets and therapeutic strategies based on autophagic resistant cells is presented.

## Introduction

**Figure 1:**
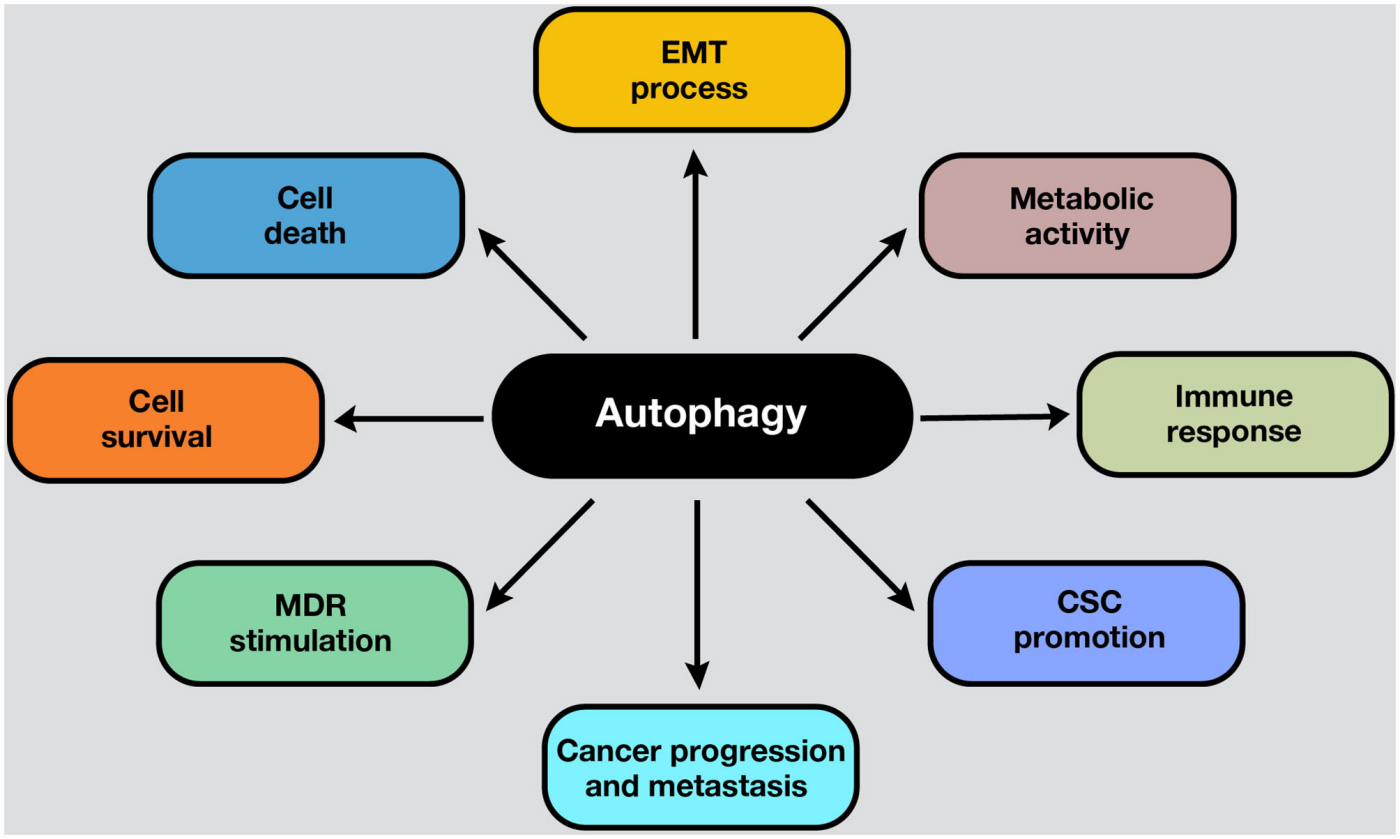
Roles of autophagy in cancer. Autophagy mechanisms are involved in several hallmarks in cancer cells. Autophagy can lead to cell survival or death, depending on the presence, duration, and intensity of the stimulus in which it develops. In addition, autophagy can modulate the EMT phenotype after the adaptation to hypoxia. Moreover, the metabolic switch of cancer cells into aerobic glycolysis (i.e., the Warburg effect) is sustained by autophagy activation, ensuring energetic requirements, and metabolic homeostasis. On the other hand, the activation of autophagy process influences the suppression or activation of antitumor immune response, depending on the stage, genetic, and microenvironmental conditions. For example, in response to chemotherapy, autophagy-competent cancer cells attracted dendritic cells, and T lymphocytes to the tumor, activating the immune response. Moreover, autophagy activation maintains the CSC phenotype and functions inside the tumor. Also, an upregulated autophagic activity are involved in cancer progression and metastasis. Furthermore, autophagic machinery triggered by anticancer drugs may facilitate multiple drug resistance in cancer cells and tumor survival. All these processes depend on the cell type, genetic background, and the microenvironment stimulus in the tumors.

Autophagy is a conserved catabolic process that sequesters and degrades intracellular components in double-membraned compartments known as autophagosomes, playing a key role in homeostasis maintenance (1). The recycling capabilities of this process prevent the accumulation of damaged proteins and organelles that can generate cell toxicity; therefore, autophagy functions as an internal quality control system (2). Autophagy is tightly regulated and normally induced in response to different intrinsic and extrinsic signals, such as starvation, growth factor deficiency, hypoxia, and many other types of stress (3). In normal conditions, the functions of autophagy comprise cell survival control to regulate homeostasis. However, in cancer cells, autophagy is frequently deregulated in and becomes important in tumorigenesis (4). Moreover, autophagy plays a pivotal role in some cancer hallmarks, including cell survival, cell death, deregulation of metabolism, modulation of the immune response, epithelial–to-mesenchymal-transition (EMT) process, cancer stem cell (CSC) promotion, and multidrug resistance (MDR) (Figure 1).

This paradoxical dual role in stimulating cell survival or promoting cell death is still under investigation in cancer at clinical and molecular levels (5). Deciphering in which genetic background and under which circumstances autophagy stimulates or eliminates cancer cells may facilitate the development of specific therapeutic strategies. Also, many studies associate autophagy with drug resistance (6). In this review, we discuss how the role of autophagy associated with cell survival and drug resistance might determine an effective therapeutic approach against, particularly aggressive tumors.

## Mechanisms of Autophagy

While the molecular mechanisms that govern autophagy in normal and cancer cells have not been thoroughly elucidated, several pathways are involved in each case. It is known that the central pathway governing autophagy is led by PI3K/AKT/mTOR signaling (7). Strikingly, this pathway is one of the most altered pathways in cancer (8, 9). The mammalian target of rapamycin (mTOR) is a highly conserved serine/threonine kinase, part of the mTOR complex 1 (mTORC1), in which different stimuli converge, including autophagy-stimulating signals (nutrient or growth factor deprivation, hypoxia, oxidative stress, or protein aggregation) (10). The activation of mTOR by growth factors exerts a negative effect on autophagy, inhibiting the autophagy process (11, 12). The process of autophagy is divided into five phases: initiation, phagophore nucleation, elongation and autophagosome formation, autophagosome-lysosome fusion, and cargo degradation, where autophagy-related genes (ATGs) play an important role in the entire pathway (13). In the initiation phase, mTORC1 is inactivated in response to these autophagy signals, and consequently, the Unc-51-like kinase 1 (ULK1 or ATG1) complex, which consists of ULK1, ULK2, ATG13, RB1-inducible coiled-coil protein 1 (RB1CC1 or FIP200) and ATG101, is activated. This complex stimulates phagophore nucleation, activating, by phosphorylation, the components of class III phosphatidylinositol 3-kinase (class III PI3K or PI3KC3) complex, which consists of vacuolar protein sorting 34 (VPS34), ATG14, activating molecule in Beclin-1-regulated autophagy (AMBRA1), general vesicular transport factor (p115), UV radiation resistance-associated gene protein (UVRAG or p63) and Beclin-1, with the last protein acting as the scaffold. This complex activates local phosphatidylinositol-3-phosphate (PI3P) production at the endoplasmic reticulum (ER), specifically in an ER structures named the omegasome (14). Then, PI3P associates with different members of the WD-repeat protein interacting with phosphoinositides (WIPI) protein family (15). Bcl-2 is a key control protein of autophagy that interacts with Beclin-1 at the Bcl-2-homology 3 (BH3) domain to inhibit its pro-autophagic activity. Bcl-2 reduces the interaction of Beclin-1 with VPS34 and UVRAG (10). However, Beclin-1 has also autophagy-independent functions; for example, it has been described to act as a negative regulator in the execution of necroptosis (16).

Elongation of the phagophore is controlled by two ubiquitin-like protein systems. First, ATG7 and ATG10 regulate the synthesis of the ATG12-ATG5-ATG16L1 complex. WIPI proteins, specifically WIPI2, bind ATG16L1 directly, anchoring the ATG12-ATG5-ATG16L1 complex to the phagophore. This complex enhances the second system, in which ATG4B, ATG7, and ATG3 act coordinately to cleave the precursors of protein light chain 3 (LC3)-like proteins and conjugate them to phosphatidylethanolamine (PE) present in the membrane. Also, γ-aminobutyric acid receptor-associated protein (GABARAP) conjugates with PE and, as a result, is incorporated into the rising autophagosome. LC3 and GABARAP give the autophagosome the capability to attach autophagic substrates targeted by selective autophagy receptors (SARs), such as sequestosome-1 (p62/SQSTM1), before membrane sealing and complete autophagosome formation (15, 17). Ultimately, microtubule proteins facilitate autophagosome transport to the lysosomes. SNARE proteins, including syntaxin 17 (STX17) and vesicle-associated membrane protein 8 (VAMP8), facilitate autophagosome-lysosome fusion. Autolysosomal contents are degraded due to the acidic lysosomal hydrolases, and the recovered nutrients are released back and recycled by the cell, using them in new metabolic processes (10, 13, 18).

**Figure 2:**
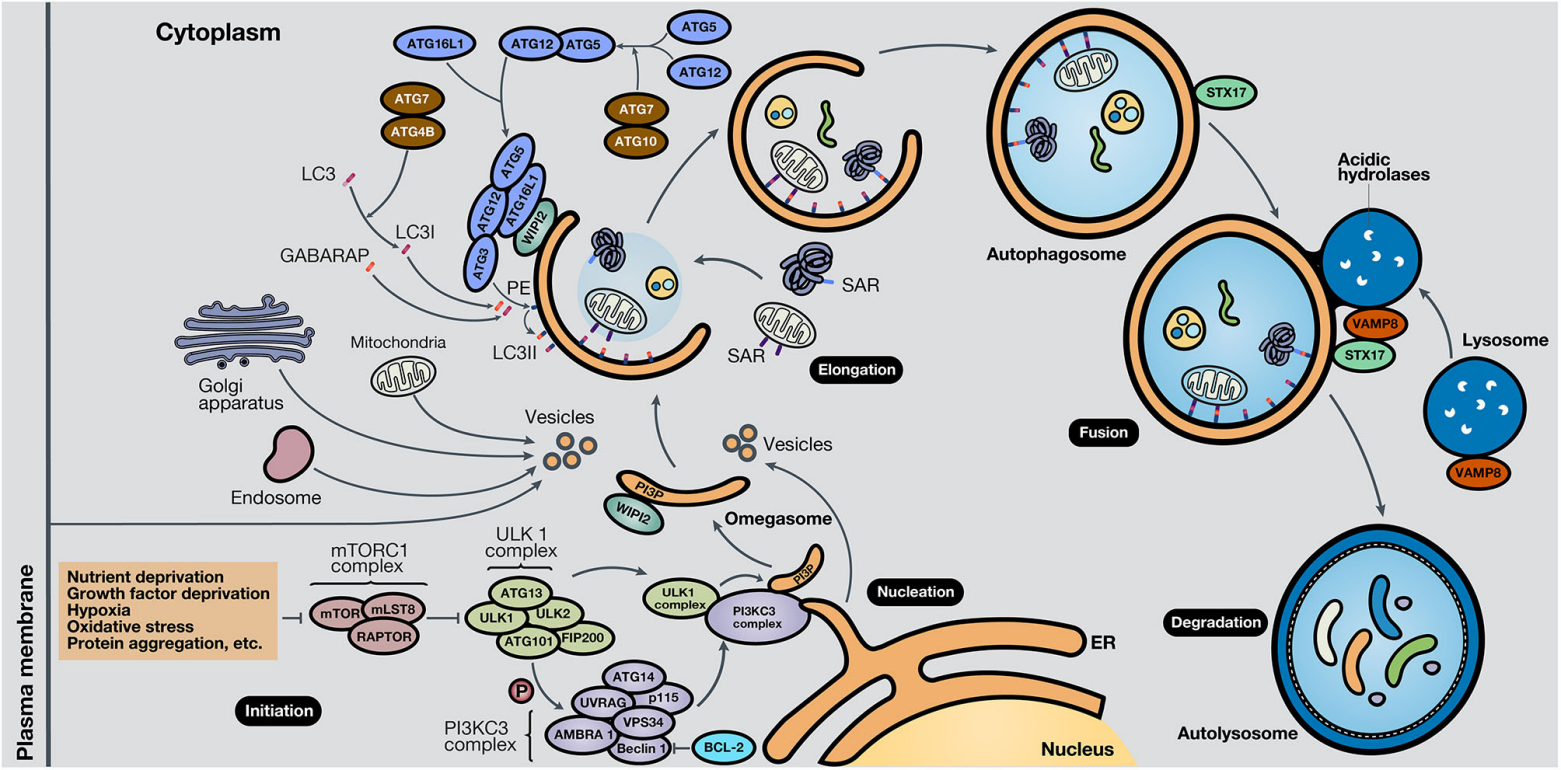
Schematic representation of the autophagy process. The autophagy mechanism consists of five phases. In the initiation phase, mTORC1 is inactivated due to autophagy-stimulating signals, liberating the repression of the ULK1 complex. During the nucleation phase, the ULK1 complex phosphorylates the PI3KC3 complex, which induces phagophore formation in the omegasome, through the production of PI3P and association with WIPI protein family members, commonly WIPI2. In the elongation phase, two ubiquitin-like protein systems, ATG12-ATG5-ATG16L1 and ATG4B-ATG7-ATG3, mediate the activation of LC3 into LC3I, lipidation with PE to form LC3II, and subsequent anchoring to the phagophore. GABARAP also conjugates with PE and attaches to the membrane. LC3 and GABARAP mediate the sequestration of autophagic substrates marked with SARs, such as p62/SQSTM1, before phagophore closure and total autophagosome development. During the fusion phase, STX17 and VAMP8, present in the autophagosome and lysosome, respectively, interact and stimulate autolysosome formation. Finally, in the degradation phase, acidic lysosomal hydrolases degrade autophagic cargo, generating nutrients that are released to the cytoplasm and reused by the cell. ER, endoplasmic reticulum.

Besides, key oncogenes inhibit autophagy, such as AKT or p21^Cip1^, while tumor suppressor genes activate it, such as PTEN, p53, and TSC1/TSC2 (19). AMPK, a protein that maintains metabolic homeostasis, is crucial for determining the destiny of autophagy. AMPK induces autophagy by phosphorylation of mTORC1, part of the mTOR pathway, and the autophagy-related complexes ULK1 and PI3KC3. Also, AMPK regulates autophagy indirectly through several transcription factors and coactivators, such as DAP1, p300, TFE/MITF, and FOXO3 (20). Proteins involved in the different phases of the autophagic process are shown in Figure 2. In the following section, the roles of autophagy in different scenarios will be discussed.

## Selective Autophagy

Selective autophagy is that type of autophagy that is specifically aimed at a specific cellular organelle. Selective autophagy is committed to preserving intracellular homeostasis by eliminating specific substrates in the autophagosome through recognition of specific receptors. Contrary to the bulk degradation process of unspecific autophagy, the objective of selective autophagy is to maintain cell homeostasis by maintaining the number of integral organelles, including mitochondria (mitophagy), ribosomes (ribophagy), aggregated proteins (aggrephagy), peroxisomes (pexophagy), lysosomes (lysophagy) or invading pathogens (21, 22). Many research findings related to selective autophagy receptors (SARs) have demonstrated that autophagy can be directed against a specific cargo. Examples of SARs, such as p62/SQSTM1, NBR1, TOLLIP, BNIP3L/NIX, and Cue5, show the mechanisms behind the formation of autophagosomes for selective autophagy (23, 24).

The cargo receptor p62/SQSTM1 is one of most extensively studied receptors and modulates selective autophagy due to its mediation in the degradation of ubiquitinated material, such as protein aggregates, mitochondria, peroxisomes, lysosomes, or intracellular bacteria (22). For example, the binding of the bacterial type III effector protein HopQ to vimentin provokes the degradation of vimentin through p62/SQSTM1-dependent selective autophagy (25). Moreover, it has been demonstrated that constant p62 levels, due to autophagy defects, were enough to alter NF-κB regulation and gene expression, thereby stimulating tumor generation (26). Another selective cargo receptor is the nuclear receptor coactivator 4 (NCOA4), which is involved in selective autophagy of ferritin, called ferritinophagy, which is activated during low levels of intracellular iron (27).

## Autophagy-Mediated Cell Death

Although it was identified as an initial function of autophagy, currently, autophagic cell death is a process that occurs less frequently in cancer cells than protective autophagy. Autophagic cell death is characterized by cytoplasmic vacuolization, accumulation, and assembling of autophagosomes labeled by LC3, and elimination of cell organelles via autolysosomes. However, the criteria to differentiate autophagic cell death from other types of cell death accompanied by autophagy are still controversial (28, 29). Although several studies suggest that an uncontrolled and nonspecific overactivation of autophagy induces cell death, other studies emphasize that the selective removal of autophagy substrates is a key factor in cell death promotion (30, 31).

Autophagic cell death—described in mammalian development, other less complex organisms, and cancer cells—can be suppressed by pharmacological or genetic inhibition or induced by specific cancer drugs (30, 32). As an example, kaempferol, a flavonoid with anticancer properties, was shown to induce autophagic cell death in gastric cancer through IRE1/JNK/CHOP signaling pathway activation, and the suppression of kaempferol-induced autophagy restores cancer cell survival (33). RY10-4, an analog version of proto-apigenone, promotes ACD by inactivation of the AKT/mTOR pathway in the breast cancer cell line MCF-7, and the inhibition of autophagy through genetic and chemical approaches extends cancer cell viability (34). Another novel anticancer drug, designated ABTL0812, which is already in preclinical trials, induces ER stress-mediated cytotoxic autophagy by increasing dihydroceramide levels in cancer cells of several models, including lung and pancreatic cancer (35). In ovarian cancer cells, activation of oncogenic H-Ras activates autophagy mechanisms, upregulating BH3-only protein Noxa and Beclin-1 and triggering cell death. Silencing of ATG5, ATG7, Beclin-1, or Noxa expression reduces autophagy and increases survival (36).

Autosis, considered a form of autophagic cell death, is regulated by Na^+^, K^+^-ATPase in the presence of Tat-Beclin-1 and Tat-vFLIP α2, Beclin-1-derived peptides, or starvation (37). Recently, treatment with Tat-Beclin-1 and Tat-vFLIP-α2 peptides showed to induce autosis as a strategy to selectively kill HIV-infected macrophage and resting memory CD4^+^ T cells, avoiding reactivation of virus (38, 39). Autosis is characterized by a dependence of Na^+^, K^+^-ATPase pump, an enhanced cell-substrate adherence, a dilated, fragmented, and finally disappeared endoplasmic reticulum, and an initial nuclear membrane convolution with a subsequent focal ballooning of the perinuclear space (37). Autosis is not entirely regulated by autophagy markers nor controlled by apoptotic and necrotic markers, although autosis is induced with a high level of autophagic activity (40). However, a recent study demonstrated the interaction of Beclin-1 and Na^+^, K^+^-ATPase, whose interaction and autotic death process increase during pathological and physiological stress conditions, and decrease by cardiac glycosides, inhibitors of Na^+^, K^+^-ATPase (41). Also, autosis can be interrupted by knockout of the autophagy-related genes ATG13 and ATG14 or by blocking treatments of autophagosomal assembly (42).

## Autophagy and Other Types of Cell Death

### Autophagy and Apoptosis

Apoptosis, a programmed cell death widely studied in cell biology, is a highly controlled process that mediates the efficient and orderly elimination of damaged cells. In the body, the balance between apoptosis and proliferation is crucial to ensure homeostasis (43). Apoptosis induces morphological changes such as cell membrane asymmetry and blebbing, protein cleavage, cell shrinkage, nuclear fragmentation, chromatin condensation, chromosomal DNA fragmentation, and phagocytic recognition (44, 45). At the molecular level, the adequate regulation of apoptosis involves several signaling pathways that control biological responses such as embryonic development, cell renewal, and external factors (e.g., radiation, chemicals), which produce DNA damage. As a result, a complete process of apoptosis implicates the interactions of many proteins, signal transducers, and signaling pathways (44). The balance between anti- and pro-apoptotic proteins is essential to decide if the apoptosis ultimately occurs. Evasion of apoptosis encourages cancer initiation and tumor progression and facilitates the emergence of resistant variants with great metastatic potential (43, 45).

**Figure 3:**
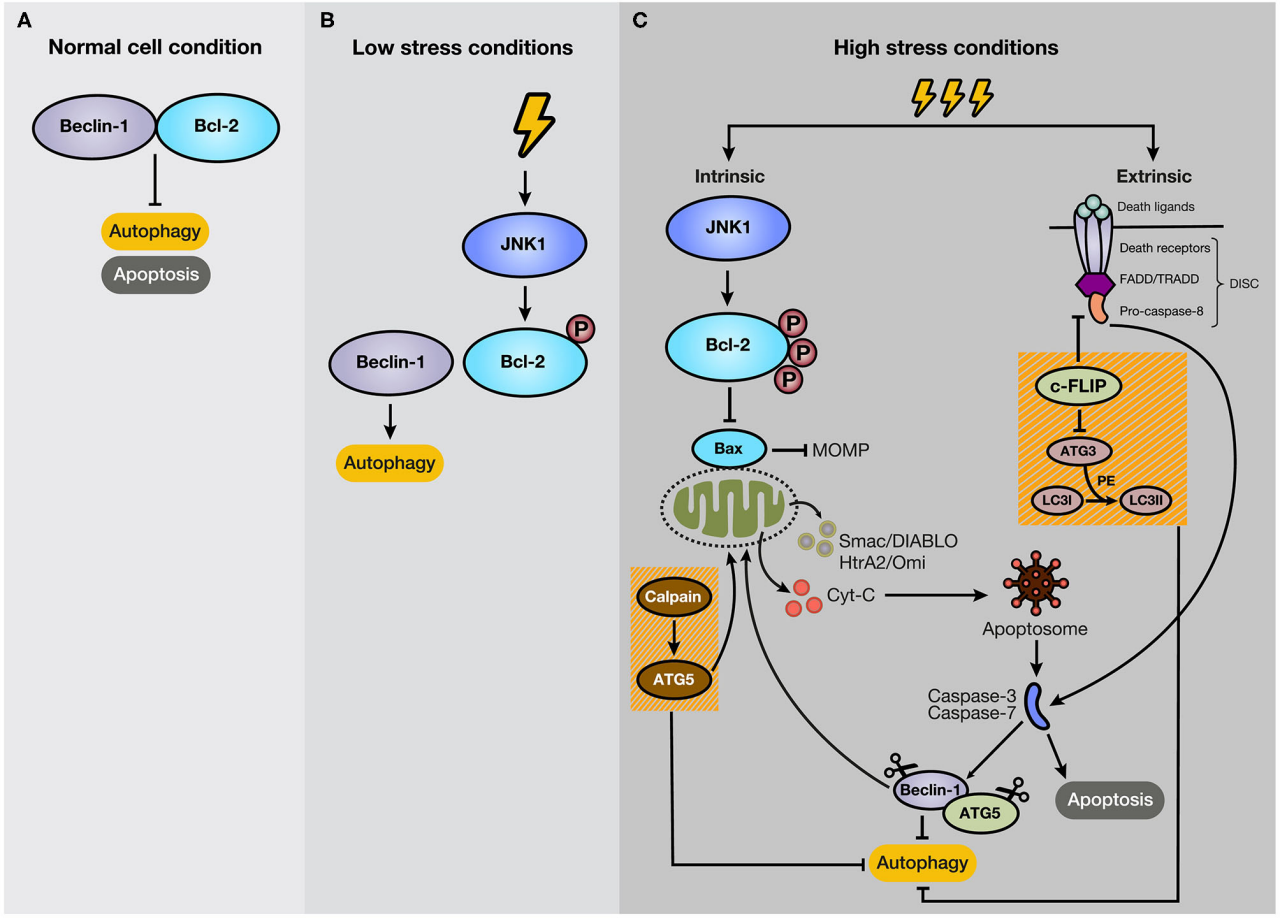
Mechanism of crosstalk between autophagy and apoptosis. **(A)** Under normal cellular conditions, Beclin-1 binds to Bcl-2, keeping autophagy and apoptosis inactivated. **(B)** However, if the cell experiences low-level stress conditions (e.g., nutrient deprivation), JNK1 phosphorylates Bcl-2, disturbing Bcl-2/Beclin-1 union. As a result, isolated Beclin-1 activates the autophagic pathway. **(C)** However, if the stressful stimulus crosses a threshold of time, JNK1 promotes Bcl-2 hyperphosphorylation, inducing its dissociation with Bax and the subsequent activation of the intrinsic apoptotic pathway. In addition, c-FLIP, a suppressor of extrinsic apoptosis, also inhibits autophagy through interaction with ATG3, reducing LC3 lipidation. Moreover, caspase activation mediates autophagy-related proteins, such as Beclin-1 and ATG5. Additionally, the C-terminal fragment generated by caspase-mediated cleavage of Beclin-1 translocates to the mitochondrial membrane and stimulates intrinsic apoptosis. Furthermore, ATG5, after cleavage by calpains, suppresses autophagy activity and induces apoptosis.

Many studies indicate that autophagy and apoptosis are closely interconnected because of their regulation by effector proteins, pathways, and intracellular locations. For example, autophagy boosts apoptosis by degrading a negative regulator of the Fas (CD95/Apo-1) ligand, but it can also protect it by modifying levels of the Bcl-2 family members. Besides, autophagy is activated by several apoptotic stimuli, and both occur after cellular stress (46). Thus, it is expected that autophagy and apoptosis in certain circumstances cooperate in cancer progression. However, the interplay of both processes is complex due to the double-edged sword function of autophagy, stimulating apoptosis, or cell survival (47). In most cases, autophagy precedes apoptosis under stress conditions. For example, low-stress conditions stimulate autophagy as a way to cope and adapt to this scenario. However, if the stress event crosses a threshold of time and/or intensity, apoptosis is activated (48). Some proteins have a dual role in apoptosis and autophagy. For example, Beclin-1 binds to Bcl-2, forming a complex at normal conditions, resulting in the inhibition of autophagy, without losing anti-apoptotic capacities of Bcl-2 (49) (Figure 3A). Bcl-2 is a mitochondrial membrane protein belonging to the Bcl-2 family, which consists of ~25 proapoptotic (e.g., Bax, Bak, and PUMA) and antiapoptotic (e.g., Bcl-2, Bcl-X_L_, and MCL-1) protein family members (50). Bcl-2 promotes anti-apoptotic functions through the interaction with Bax, which repress the Mitochondrial Outer Membrane Permeabilization (MOMP) (51), and the subsequent release of proteins, such as cytochrome c (cyt-c), high-temperature requirement protein A (HtrA2/Omi), and second mitochondria-derived activator of caspase/direct inhibitor of apoptosis (IAP)-binding protein with low pI (Smac/DIABLO) to the cytosol (52). In cancer cells under starvation, C-Jun N-terminal protein kinase 1 (JNK1) becomes activated and phosphorylates Bcl-2, disrupting the Bcl-2/Beclin-1 complex and promoting autophagy due to the activation of core autophagic components by isolated Beclin-1 as a response for cell protection (47, 53) (Figure 3B). However, if the starvation is prolonged, JNK1 induces hyperphosphorylation of Bcl-2 that generates its dissociation with Bax and apoptosis stimulation (54) (Figure 3C). Therefore, Bcl-2 and Beclin-1 interaction represent a significant mechanism for regulating the switch between autophagy and apoptosis. As an example of other members of Bcl-2 family, BNIP3 and NIX are also implicated in the stimulation of autophagy, and specifically mitophagy, due to a BH3 domain in their structure, apart from their role as pro-apoptotic proteins (55, 56). The augmentation of reactive oxygen species (ROS) production and the competition for Bcl-2 binding with Beclin-1, with consequent Beclin-1 liberation, are strategies that BNIP3 and NIX can apply to induce autophagy (56). Moreover, BNIP3 and NIX regulates mitophagy through HIF-1α/BNIP3 signaling pathway, which promotes a decrease of ROS production and plays a protective role during hypoxia (57–59).

Beyond their autophagic functions, many autophagy-related proteins have a pivotal role in apoptosis. For example, non-conjugated forms of ATG5 and ATG12 induce apoptosis under stress conditions. ATG12 directly binds to Bcl-2 family members, including the antiapoptotic proteins Bcl-2 and MCL-1, independent of its interaction with ATG5 or ATG3 (60). ATG5 is cleaved by calpains, suppressing its autophagy activity (Figure 3C). Also, the N-terminal fragment of ATG5 translocates to mitochondria and induces the release of cyt-c, leading to the activation of effector caspases and apoptosis (61). Some studies indicate that overexpression of ATG5 sensitizes tumor cells to chemotherapy, and knockout of this protein increases tumor cell resistance to chemotherapeutic drugs (62, 63).

Additionally, some key apoptotic proteins also participate in the regulation of autophagy. For example, FADD-like IL-1β-converting enzyme-inhibitory protein (c-FLIP) is an anti-apoptotic protein that suppresses extrinsic apoptosis (62). Ligation of dead receptors, such as type 1 TNF receptor (TNFR1), Fas, and TRAIL, at their extracellular domain generates recruitment of specific procaspases (−8 and sometimes −10) and adaptor proteins to the cytosolic domain, such as Fas-associated death domain (FADD) and TNFR-associated death domain (TRADD), forming a multiproteic structure called death-inducing signaling complex (DISC) (64). Due to the death effector domain (DED) present in its structure, c-FLIP interferes with the interaction of dead receptors, and adaptor proteins (50). Besides, FLIP suppress autophagy through blockage of LC3 lipidation by competitive interaction with ATG3 (Figure 3C). In contrast, if the autophagic process is initiated, the FLIP and ATG3 interaction is substantially reduced (65).

Autophagy usually becomes regulated due to cleavage of essential proteins in the autophagic process by caspases (48). Caspases (cysteinyl, aspartate-specific proteases) comprise a family of cysteine proteases that mediate the molecular process of apoptosis and participate actively in the initiation and execution pathways (66). Caspases are crucial proteins in the apoptosis process and are involved as apoptotic initiators (caspase-2,−8,−9, and−10) and executors (caspase-3,−6, and−7) of cell death (67). Essential autophagy proteins, such as Beclin-1, ATG3, ATG5, and ATG7, are cleaved by caspase-3,−7, and−8, destroying their autophagic function (68). Also, caspase-mediated cleavage of Beclin-1 produces a C-terminal fragment that translocates to mitochondria and boosts intrinsic apoptosis (69, 70) (Figure 3C). However, although caspase cleavage of ATG4, principally ATG4D by caspase-3 (71), induces cytotoxicity through its movement to the mitochondria, this autophagy-related protein also induces the autophagy pathway (29), demonstrating a complex interaction with very fine regulation determined by the levels of apoptotic and anti-apoptotic proteins present in the cells.

### Autophagy and Necroptosis

Necroptosis was discovered as a new form of strictly regulated programmed cell death with characteristics of necrosis (72). Escape from necroptosis via loss of RIPK3 expression is a feature of some cancers. Moreover, downregulation of necroptosis mediators such as RIPK3 and MLKL in tumors suggests an escape mechanism from necroptosis in cancer (73). Necroptosis is principally controlled by receptor-interacting protein kinase 1 (RIP1 or RIPK1), RIPK3, and mixed lineage kinase domain-like pseudokinase (MLKL), and its activation is mediated by death receptors, mainly TNFR1 (74). Death receptor binding with its ligand, tumor necrosis factor α (TNFα), promotes the recruitment of RIPK1, TRADD, a cellular inhibitor of apoptosis protein 1 (cIAP1), cIAP2, TNFR-associated factor 2 (TRAF2), and TRAF5, forming pro-survival complex I. RIPK1, which is polyubiquitinated in complex I, assembles complex IIa after deubiquitination, formed by RIPK1, RIPK3, TRADD, FADD, and caspase-8. Complex IIa mediates caspase-8 activation and subsequent apoptosis. However, if caspase-8 is inhibited, RIPK1 recruits RIPK3, forming complex IIb that, after their phosphorylation, activates the necroptosis pathway through the establishment of a necrosome (75). Then, RIPK3 phosphorylates MLKL, promoting its oligomerization and translocation to the plasma membrane, which boosts membrane permeabilization due to phospholipid disturbance. This stimulation of membrane permeability, resulting in cytokine and chemokine release, causes an immune response that provokes inflammation and determines the outcome of apoptosis or necroptosis (76).

Necroptosis and autophagy maintain a close and complex interplay, considering that both processes can be activated sequentially or at the same time, activating or suppressing one with the other, and with the same or contrary purposes of cell survival or death (77). Regularly, autophagy is activated to restore levels of energy, saving cells that would otherwise undergo necroptosis due to ATP deficiency (78). Besides, autophagy is induced by necroptosis as a reaction to high levels of reactive oxygen species (ROS) produced, eliminating critically damaged cell structures, ensuring homeostasis, and ultimately avoiding necroptotic cell death (79). Phosphorylation of VSP34 and Beclin-1 by protein kinase D1 (PKD) and death-associated protein kinase (DAPK), respectively, to stimulate autophagosomal formation are two examples of autophagy activation mechanisms against oxidative stress, with subsequent necroptosis suppression (77). Another example is the induction of necroptosis signaling by poly(ADP-ribose) polymerase-1 (PARP-1) overactivation, which provokes ATP depletion and consequent autophagy pathway activation through the LKB1-AMPK-mTOR pathway to ensure cell survival (80). Therefore, autophagy inhibition during low cell energy availability could generate a metabolic crisis that promotes necroptosis activation (78).

Several studies highlight the caspase-8/RIPK1 interaction as crucial in the regulation of the autophagy pathway and the interplay between autophagy, necrosis, and apoptosis (74). For example, caspase-8 is triggered inside autophagosomal membranes in some cases and acts as a platform and eliminates inhibitors of apoptosis, promoting apoptosis (81). Also, activated caspase-8 cleaves RIPK1, reinforcing apoptotic vs. necroptosis signaling. However, in a MAP3K7 deletion context, autophagy changes the death cell mode toward necroptosis, recruiting and scaffolding RIPK1 via p62/SQSTM1 to the autophagosome. As a result, the necrosome becomes more selectively and quickly activated (82).

Moreover, necroptosis and autophagy can be activated in parallel to boost cell death (78). For example, some investigations with zVAD, a general caspase and apoptosis inhibitor, evidenced a stimulation of necroptosis and autophagy by this peptide after TNFα stimulation, characterized by the formation of autophagosomal vacuoles (77). However, the cell death response can be suppressed by downregulation of RIPK1, ATG7, or Beclin-1 expression (83). Thus, these autophagic genes participate actively in the control of necroptosis-mediated cell death.

### Autophagy and Pyroptosis

Pyroptosis is a regulated cell death accompanied by a proinflammatory response. Various microbial infections and internal damage-associated signals, such as dysfunctional mitochondria, induce the assembly of inflammasome, a multiprotein complex that promotes the activation of inflammatory caspases (-1,−4,−5, and−11), which mediate the pyroptotic signaling pathway (84). These non-apoptotic caspases play two important roles in pyroptosis activation. First, inflammatory caspases activate the inflammatory cytokines interleukin 1β (IL-1β) and IL-18 (75). Second, caspases activate Gasdermin D (GSDMD), a pyroptotic protein that, after caspase-mediated cleavage of its N-terminal fragment (GSDMD-N), moves toward the inner plasmatic membrane by generating porosity and permeabilization (85). It results in an uncontrolled flow of ions and water, causing cell lysis, cell death, and subsequent release of additional cytokines in the extracellular microenvironment (86). Pyroptosis, contrary to apoptosis and other types of cell death, is characterized by maintaining nuclear integrity, without DNA fragmentation, but showing signs of nuclear condensation and cell swelling (75, 85).

The autophagy mechanism plays an important role in the suppression of pyroptosis by inactivation of the inflammasome (87). To avoid the pyroptotic pathway, autophagy applies two strategies. First, autophagy sequestrates inflammasome inducers such as ROS, bacteria, and critical damaged mitochondria that, after ubiquitination for recognition, are delivered to autophagosomes for degradation (24). Second, autophagic machinery recognizes overactivated components of the inflammasome, especially NLR family pyrin domain-containing protein 3 (NLRP3) and Absent In Melanoma 2 (AIM2), which are specifically recognized by the autophagy receptor p62/SQSTM1, transported and destroyed via the autophagosome (88, 89). Both strategies limit the activation and release of the proinflammatory cytokines IL1β and IL-18, reducing inflammation and pyroptosis signaling (87, 89).

### Autophagy and Ferroptosis

Ferroptosis is a novel type of programmed cell death characterized by iron and lipidic ROS/peroxides accumulation (29). It has been proposed that cancer cells from different tissues show different degrees of ferroptosis sensitivity. Even so, some authors have shown that ferroptotic reagents can induce cancer cell death that could be rescued by ferroptosis inhibitors (90). This iron- and oxidative-mediated cell death is activated through excessive levels of iron production by Fenton reaction and through the loss of balance in ROS production and cell glutathione (GSH)-dependent antioxidants, which protect cells from lipid peroxidation (85). Glutathione peroxidase 4 (GPX4) is a crucial enzyme for the elimination of lipid ROS continuously generated by the cell. Its inhibition can induce ferroptosis even with normal levels of the cofactor GSH (91). Besides, depletion of GSH or its precursor, cysteine (Cys), constitutes an indirect way to activate ferroptosis (92). Ferroptosis is characterized, contrary to other regulated cell death mechanisms, by cell membrane integrity, normal nucleus size, and dense small mitochondria (76).

Recent studies have described a direct contribution of autophagy in ferroptosis initiation, arguing the presence of a specific autophagic cell death called ferritinophagy (91). After Cys suppression, autophagy is activated to sequester and degrade ferritin, a cell iron storage protein, by the selective autophagy cargo receptor NCOA4, inducing ROS accumulation and the consequent ferroptotic cell death. Inhibition of the expression of autophagic proteins such as ATG5, ATG7, and NCOA4 reduces ferritin elimination, iron levels, lipid peroxidation, and ferroptosis activation (93). Furthermore, autophagy pathway activation by Tat-Beclin-1, a direct autophagic-mechanism inducer, selectively promotes ferroptotic cell death in tumor cells (94). Other studies demonstrate that ferroptosis stimulation also induces autophagy, evidenced by an intensification in the conversion of mature LC3 and autolysosome assembly (95), demonstrating a close interplay between both signaling mechanisms.

## Autophagy and EMT

The epithelial-to-mesenchymal transition (EMT) is a key process involved in the genetic, biochemical, and phenotypic changes that epithelial cells experience to convert them to mesenchymal cells, a cellular type with greater versatility and plasticity (96). Further, has been discovered that the reverse process, designated mesenchymal-to-epithelial transition (MET), is also crucial in the metastatic process. When a cell undergoes EMT, it loses its basal polarity to acquire a fibroblast-like morphology (97). EMT is important to allow migratory properties to the cancer cells, facilitating their entry into the bloodstream. Further, to find a niche in any tissue, the cancer cells need to exit from the circulation by experiencing the MET process to acquire epithelial properties to nest in the tissues and establish metastatic niches. The most important signals to stimulate EMT occur in the microenvironment. For example, hypoxia, which appears in certain parts of the tumor that are oxygen-deprived, generates EMT through by the activation of HIF-1α, which in turn stimulates inflammatory cytokines (e.g., TNFα or IL-6), which contribute to EMT induction (98, 99). Moreover, hypoxia induces high levels of ROS in cancer cells, which leads autophagy stimulation (100, 101). Conversely, autophagy also increases the EMT phenotype after the adaptation to hypoxia (102).

The effect of autophagy on EMT appears controversial and depends on the type of stimulus, the cell genetic background, and the cell type. Different cytokines or microenvironmental conditions that stimulate EMT can provoke opposite reactions in autophagy (97). For example, salvianolic acid B, an active component of a Chinese natural product, suppresses EMT in a renal fibrosis animal model by induction of autophagy, mediated by silent information regulator 1 (Sirt1) (103). Imprinted gene pleckstrin homology-like domain family A member 2 (PHLDA2) is upregulated in colorectal cancer, and its knockout stimulates autophagy via the PI3K/AKT pathway, reducing cell proliferation, invasion, migration, and EMT process (104). In gastric cancer, forkhead box K1 (FOXK1) is a transcription factor involved in cancer development. The inhibition of FOXK1 in an acidic microenvironment triggers autophagy and reverses EMT in gastric cancer cells (105). However, in bladder cancer cells, it has been demonstrated that starvation conditions promote autophagy, which boosts the EMT process through TGF-β1/Smad3 signaling, enhancing cell invasion and migration (106). Moreover, knockdown of the autophagy-related protein DNA damage-regulated autophagy modulator 1 (DRAM1) reduces the migrative and invasive capabilities of hepatoblastoma cells, inactivating autophagy, and EMT (107).

## Autophagy and Metabolism

It has been assumed that malignant cells have a hyperactivation of metabolic activities that increase ROS levels. However, the known Warburg effect described one century ago in cancer cells, is based upon the use of glycolysis, even in the presence of oxygen, to avoid the OXPHOS respiration through the mitochondria and, consequently, high ROS accumulation (108, 109). The high use of glycolysis generates huge concentrations of lactic acid released in the microenvironment. It has been suggested that not only the avoidance of ROS accumulation gives an extra survival capacity to cancer cells but also the lactic acid acidifying the microenvironment (110). For example, it has been described that in melanoma cells, glucose-deprivation stress induces autophagic cell death, but this is inhibited by the large concentrations of lactic acid in the microenvironment (111).

Autophagy can be activated by ROS through diverse signaling pathways, such as ROS-FOXO3-LC3/BNIP3, ROS-NRF2-P62, ROS-HIF1-BNIP3/NIX, and ROS-TIGAR; as a result, autophagy suppresses ROS-promoted damage by eliminating oxidized substance, keeping cellular homeostasis (112, 113). In cancer, autophagy also regulates tumor homeostasis, preventing the accumulation of ROS generated by the hyperactivation of metabolism (114). On the other hand, in principle, autophagy counteracts the metabolic switch followed by malignant transformation by eliminating deteriorated mitochondria to sustain the maximum bioenergetic needs and preserve the physiological, metabolic homeostasis. ROS has been described to oxidize ATG4, resulting in the formation of autophagosomes and autophagy (115). This process occurs in cadmium-mediated cell proliferation, migration, and invasion in pulmonary adenocarcinoma cells (116).

Moreover, ATG12 has been shown to control mitochondrial biogenesis and metabolic pathways such as glycolysis, tricarboxylic acid cycle, and β-oxidation in cancer cells (117). Additionally, tyrosine kinase signaling by hepatocyte growth factor (HGF) and its receptor tyrosine kinase (MET/HGFR) is hyperactivated in numerous cancers, inducing proliferation, invasion, and metastasis. In liver cancer, HGF/MET pathway activation provokes the Warburg effect and glutaminolysis, mediating cancer cell development. However, targeting MET to suppress kinase activation triggers the autophagy pathway to ensure cell growth and survival (118). In nasopharyngeal carcinoma, the Epstein-Barr virus latent membrane protein 1 (LMP1) can promote tumor development by its transference inside extracellular vesicles released by fibroblasts, boosting their transformation into cancer-associated fibroblasts (CAFs) via the NF-κB pathway. As a result, CAFs activate autophagy machinery and mediate a metabolic switch from OXPHOS to glycolysis to generate energy-rich nutrients for cancer cells, which enhance their OXPHOS metabolic activity, in a process called the Reverse Warburg Effect (RWE) (119).

## Autophagy and the Immune System

Autophagy participates actively in the regulation of the immune system, playing significant roles in the activation, differentiation, and survival of immune cells such as T and B cells, monocytes, macrophages, natural killer (NK)-cells, and dendritic cells. Thereby, the autophagic process modulates innate and adaptative immunity (120, 121). Also, autophagy controls the production and release of cytokines, such as IL-1, IL-18, Type I IFN, and TNF-α. Apart from immune cells, immune components, such as cytokines and immunoglobulins, influence the activation and suppression of autophagic processes. It has been described that IL-1, IL-2, IL-6, IFN-γ, TNF-α, and TGF-β1 are stimulators and IL-4, IL-10, and IL-13 are inhibitors of the autophagy process (122). For example, natural secretion of IL-17 and IL-22 by γδ T cells can be regulated by IL-1-dependent autophagy activation (123). Moreover, in antigen donor cells, upon severe stress exposure (which might be prolonged in time), cell death will take place, causing autophagy-mediated antigen release and stimulation of immune and inflammatory responses (124).

Several studies indicate that a piece of active autophagic machinery produces tumor-specific antigens in tumor cells which, after their release due to antigen donor cells, boost antitumor effects by enhancing antigen presentation and subsequent T cell activation (120). Autophagy induces liberation of more ATPs as a required signal to stimulate recruitment of antigen-presenting cells (APCs) and tumor sensitivity to cytotoxic T lymphocytes (124). Also, inhibition of autophagy by targeted drugs or genetic deficiencies in autophagy-related genes such as ATG5, ATG7, ATG12, Beclin-1, and VPS34 reduces ATP release and hinders the recruitment of required immune cells from boosting antitumor immune responses (125). Furthermore, radiotherapy- or chemotherapy-induced autophagy mediates the release of the mannose-6-phosphate receptor (MPR) without its natural ligand from the autophagosome and its movement back to the cell surface, promoting T cell activation after granzyme B binding (126). Autophagy has also been involved in antigen processing for major histocompatibility complex class I (MHC-I) and II (MHC-II) presentation, including cross-presentation. To give an example, alpha-tocopheryloxyacetic acid (α-TEA), derived from vitamin E, promotes autophagy-controlled cross-presentation of tumor antigens in lung cancer cells to the immune system, mainly antigen-specific cytotoxic T lymphocytes (127).

Moreover, autophagy can suppress immune effector mechanisms against tumors. For example, the hypoxia condition triggers autophagy machinery in lung cancer cells, which suppresses T cell antitumoral activity through phosphorylation of STAT3 and subsequent HIF-1α signaling pathway activation (128). Tumor susceptibility to the cytotoxic effect and tumor cell lysis of T lymphocytes are restored through hydroxychloroquine (HCQ)-mediated autophagy inhibition or knockdown of ATG and Beclin-1 genes (125, 129). Moreover, hypoxia-induced autophagy also interrupts the anticancer killing activity of NK-cells by selective degradation of NK-derived granzyme B, which can be reversed after autophagy inhibition by targeting Beclin-1 (130, 131). On the other hand, tumor-associated macrophages (TAMs) are key components of the immune system and the main drivers of inflammatory microenvironment inside tumor and cancer progression (132). According to a recent study in metastatic ovarian cancer, TAMs that specifically express T-cell immunoglobulin and mucin domain-containing 4 (TIM4) showed high oxidative phosphorylation and adapted mitophagy to mitigate oxidative stress (133). Besides, genetic deficiency of autophagy protein FIP200 ensued in Tim-4^+^ TAM loss via ROS-mediated apoptosis increasing T cell-immunity and tumor inhibition *in vivo* (133).

Therefore, autophagy activation can induce antitumor immune responses but can also mediate inhibition of immune cell activity against tumors to allow cancer cells to escape from the immune system. Overall, autophagy has a context-dependent function as an activator and inhibitor of the immune response in cancer cells, which might be crucial in current immunotherapies.

## Autophagy and Non-Coding RNAs

Non-coding RNAs (ncRNAs) comprise 98% of the human genome, and their biological functions consist of chromatin and epigenetic modifications, regulation of gene expression, transcription, mRNA splicing, regulation of protein localization and activity, and apoptosis, among others (134). These regulatory RNAs are classified into two groups: long ncRNAs (lncRNAs), larger than 200 nucleotides, and small ncRNAs, which mainly comprise microRNAs (miRNAs), small interfering RNAs (siRNAs), small nuclear RNAs (snRNAs), small nucleolar RNAs (snoRNAs), circular RNA (circRNAs), and piwi-interacting RNAs (piRNAs) (135). The role of ncRNAs in cancer cells has been associated with many physiological and pathological processes, such as proliferation, differentiation, migration, invasion, metastasis, and drug resistance (136).

Recent studies have described the mechanisms of several ncRNAs in the regulation of the autophagy process in tumor cells (137). For instance, circNRIP1 was proven to modulate the autophagy and cancer cell metabolism switch into the Warburg effect by alteration of AKT1 expression and, consequently, the AKT/mTOR pathway, which induces tumor development and metastasis in gastric cancer (138). Moreover, miRNA-133a-3p suppresses tumor growth, and the development of metastatic lesions in gastric cancer, inhibiting autophagy-mediated glutaminolysis by targeting GABARAPL1 (a GABARAP subfamily) and ATG13 (139). Additionally, miR-142-3p was demonstrated to target ATG5 and ATG16L1, causing the inhibition of autophagy, producing an increased sensitization of hepatocellular carcinoma cells to sorafenib (140). Also, miR-519a sensitizes glioblastoma cells to temozolomide by the activation of autophagy via the STAT3 pathway, which generates Bcl-2/Beclin-1 complex dissociation and resultant autophagy-mediated apoptosis (141). There are many other miRNAs, such as miR-124, miR-144, miR-224-3p miR-301a/b, and miR-21, involved in the alteration of autophagy in many cancer cell types, either activating or inhibiting, which influence tumor resistance to conventional therapy (142–145). Additionally, lncRNAs control autophagy mainly by directly or indirectly regulating ATG expression (146). As an example, knockdown in colorectal cancer cells of homeobox transcript antisense intergenic RNA (HOTAIR), a lncRNA that has been widely studied, induces upregulation of miR-93 and a downregulation of ATG12, resulting in a blockage of autophagy and the induction of apoptotic cell death (147). In hepatocellular carcinoma, the lncRNAs phosphatase and tensin homolog pseudogene 1 (PTENP1) activate autophagy, interacting with miR-17, miR-19b, and miR-20a, denying their targeting of the autophagy genes ULK1, ATG7 and p62/SQSTM1, and the tumor suppressor PTEN. As a result, the overexpression of PTENP1 reduces tumor size, restrains proliferation, suppresses angiogenesis, and induces cancer cell apoptosis (148). Also, highly upregulated lncRNA in hepatocellular carcinoma cells diminishes their sensitivity to chemotherapeutic drugs by autophagy triggering, mediated by suppressing silent information regulator 1 (Sirt1) (149). Other lncRNAs, such as XIST, BLACAT1, and MEG3, also play a pivotal role in the regulation of autophagy processes in different types of tumors, which modulate cancer progression and chemotherapeutic resistance (150–152).

## Autophagy and CSCs (Cancer Stem Cells)

The cancer stem cell hypothesis proposes that many cancer types originate from cancer cells with stemness-like characteristics, known as cancer stem cells (CSCs) (153). CSCs are a subpopulation of cancer cells that possess the abilities of differentiation, tumor initiation, pluripotency, and self-renewal capabilities, being able to reconstruct the original tumor by themselves. CSCs are the cell type most representative of resistance to conventional anticancer therapies (including radiation and chemotherapy) in comparison to other cells that constitute the tumor (154). These features confer CSCs the abilities of tumor relapse and metastasis dissemination. Besides, CSCs show the capacity to grow under serum starvation, forming spheres in 3D conditions, maintaining high aldehyde dehydrogenase (ALDH) activity while showing cell cycle dysregulation (155). Moreover, under the term CSCs, there is a large heterogeneous population of different CSCs with different degrees of malignancy (156).

Many studies underline the crucial role of the autophagy mechanism in the maintenance of CSC homeostasis, features, and functions inside tumor and cancer progression (157). CSCs use autophagy to reinforce their resilience against microenvironmental stress conditions, such as starvation and hypoxia, promoting their survival to preserve their stemness phenotype (155). It has been proposed that through TGF-β1 inducing EMT in CSCs; it is autophagy activation that enables them to invade the circulation. In breast cancer, the inhibition of the autophagy-related proteins Beclin-1, ATG12, and LC3 reduces the stemness-like phenotype, reinforcing that the activation of protective autophagy supports the maintenance of the breast CSC population (158, 159). In the same line of evidence, autophagy inhibition by knockdown of ATG5 and ATG7 drastically decreases the stemness features of colorectal CSCs, evidenced by a reduction in the expression levels of stemness markers (OCT4, SOX2, and NANOG), increased cellular senescence, and the decline of cell proliferative capacities in CSCs in tumors (160). As would be expected, enhancement of the autophagy pathway in colon cancer induces resistance to anticancer therapies and an increase in the stemness-like phenotype (161). In glioblastoma, the autophagy regulator p62/SQSTM1 and DNA damage-regulated autophagy modulator 1 (DRAM1) is highly expressed in CSCs and control their migrative and invasive capacities (162, 163).

Additionally, some studies associate pluripotency-related factors with autophagy activation (55). For example, in non–small lung carcinoma, melanoma, and breast cancer, NANOG induces autophagy under hypoxia conditions in CSCs by direct regulation of BNIP3, a protein that interacts with Bcl-2 and mediates the disruption of the Bcl-2/Beclin-1 interaction (164), promoting tumor cell immune resistance (165). Furthermore, SOX2 induces autophagy through enhancement of ATG10 gene expression in colon cancer cells (165). These results corroborate that autophagy is an essential process involved in stem-like phenotype maintenance and tumor resistance to treatment in CSCs.

## Autophagy in Stress Responses, Cancer Progression, and Metastasis

It is broadly known that a basal level of autophagy is present in all cell types that naturally occurs. In contrast, an increase of the autophagy pathway or the autophagy flux accounts when cells are exposed to certain levels of stress (166). The autophagic stress response consists of two parts: a very rapid increment (minutes or hours after exposure to the stressor) in the autophagic flux through post-translational modifications, and a long term autophagic response consisting in the activation of stress-responsive transcription programs, being transcription factors such as p53, NF-κB, and STAT3 relevant in regulation of the autophagy facing stressful conditions (167).

As we analyze before, autophagy activity can be tumor suppressive or promoting depending on the scenario, such as nutrient availability, microenvironment influence, immune response, and among others (168). Genomic analysis of human cancers has identified that oncogenic events involving classical oncogenes and tumor suppressor genes have a key role in autophagy including PI3K, AKT1, PTEN, proteins of the Bcl-2 family, among others (169). However, functional evaluation of autophagy at the clinical level is demanding because the autophagic flux is not possible to measure in tumor samples of patients (168, 170). Even so, different studies corroborate that autophagy is upregulated in different types of cancer since progression to metastasis, and expression of several autophagy markers has been correlated with poor outcomes (171). As an example, the identification of a novel autophagy associated-gene signature can predict the prognosis of cancer patients with hepatocellular carcinoma. Such five genes are HDAC1, RHEB, ATIC, SPNS1, and SQSTM1, that were associated with overall survival in hepatocellular carcinoma patients (172). Of interest, the expression of autophagy-related genes was correlated with drug sensitivity in hepatocellular carcinoma cell lines (172).

Autophagic activity plays the primary role in the regulation of the different metastatic phases, including invasion, intravasation, survival inside the circulation, extravasation, survival, and growth in the second site; and also in the diverse mechanisms involved in metastasis, such as focal adhesion, integrin trafficking, cytoskeleton remodeling, anoikis resistance, detachment from the extracellular matrix, EMT, and tumor-stromal interaction (170, 173). Although it is challenging to determine autophagy flux in tumor patients, surrogate markers, such as LC3, have found a correlation between increased levels of autophagy and metastasis generation in varied types of cancer (112, 174, 175). Moreover, novel proteins related to metastasis have been shown to have a role in autophagy. For example, Nuclear protein 1 (NUPR1) is a molecule regulated in response to stress, that has been implied in the progression of many cancers including of breast, pancreas, brain, and thyroid, in the development of metastasis (176, 177). NUPR1, initially associated with the rescue of cells to doxorubicin-induced genotoxic stress, has been shown to have a multifaceted role, including involvement in autophagy (178). Chaperones represent other examples. BAG3, a multifunctional HSP70 co-chaperone, exerts various physiological functions, including stress response and apoptosis, and oncopathological roles such as cell adhesion, metastasis, angiogenesis, stimulation of autophagy flux, and others. Also, BAG3 interacts with HSP70 and LC3 delivering polyubiquitinated proteins to the autophagy pathway. (179).

## Autophagy and Cancer Cell Resistance

Intriguingly, different chemotherapeutic drugs may exert opposite effects on autophagy, resulting in cell death or cell survival. Autophagy in cancer cells during stress might emerge spontaneously due to gene mutations/epigenetic modifications or due to an imbalance of the cellular capacity to control its growth during adverse conditions. Moreover, ribosomal stress, ER stress, or the unfolded protein response (UPR) can trigger autophagy (180). In the last decade, the role of autophagy has been reinforced as a protective mechanism to mediate cell survival during chemotherapy, conferring MDR (181). For example, ATP-binding cassette (ABC) transporters, specifically ABCB1, also known as multidrug resistance protein 1 (MDR1), have been associated with MDR against a wide variety of chemotherapeutic agents (6). The expression in ABCB1 is positively correlated with autophagic-related genes Beclin-1, LC3, Rictor, and poor outcome survival of colorectal cancer patients (182), highlighting an association between autophagy triggering and MDR. It has been demonstrated that resistance to FGFR1-targeted therapy promotes autophagy via the TAK1/AMPK pathway (183).

**Figure 4:**
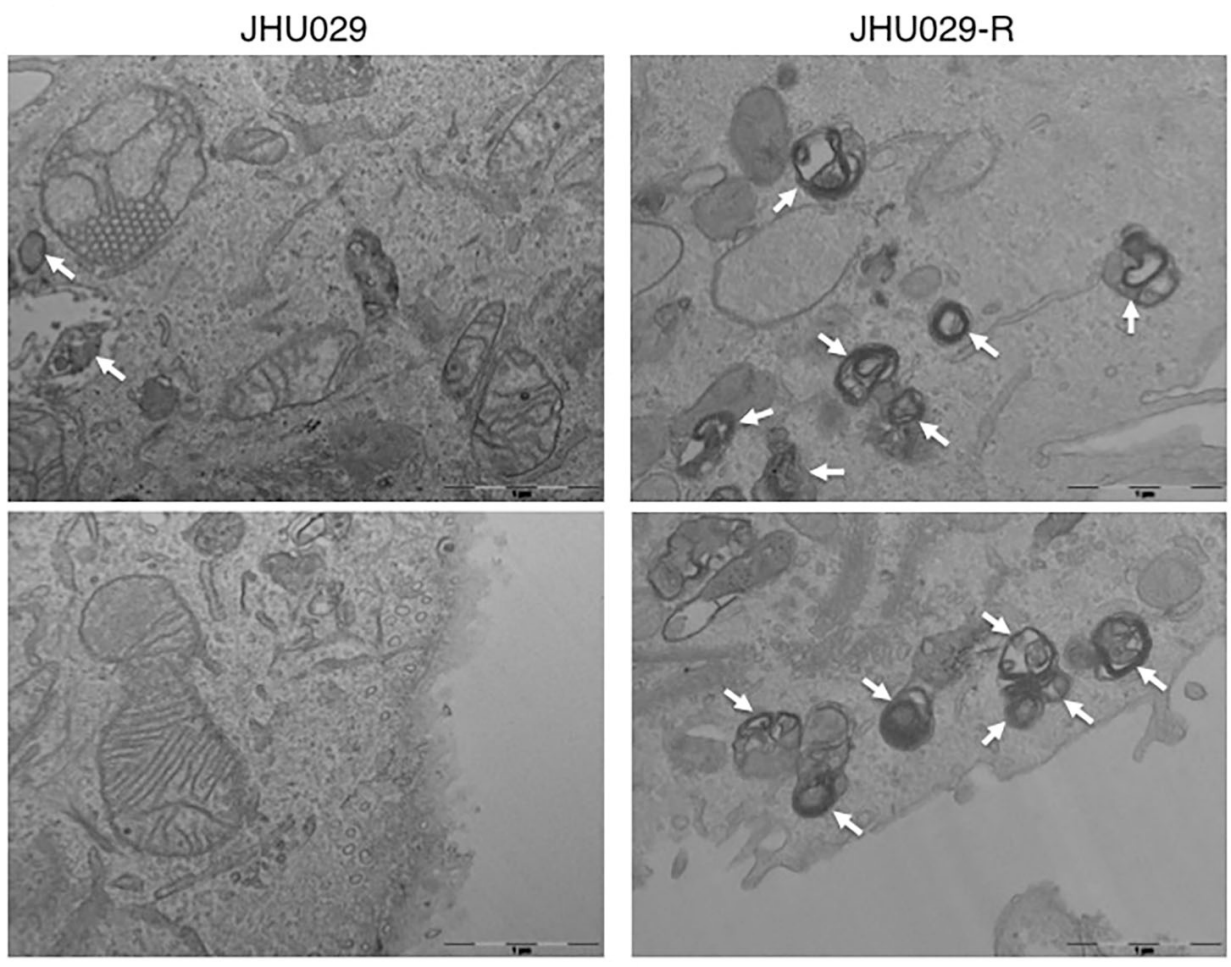
Autophagy vesicles in cancer cells sensitive and resistant to chemotherapy. TEM images showing the presence of autophagy vesicles in JHU029 cell lines derived from laryngeal cancer, sensitive, and resistant (R) to cisplatin (see arrows). As was demonstrated by our group (187), cancer cells resistant to chemotherapy generate more autophagy vesicles than sensitive ones, which is correlated with the resistant phenotype developed.

Furthermore, several studies proved that autophagy stimulated by anti-cancer drugs probably enable the development of multiple resistance feature against epirubicin, paclitaxel, tamoxifen or herceptin, through inhibition of apoptosis in breast cancer cells (184). Besides, miR-495-3p was found to regulate autophagy and, consequently, MDR by its interaction with the GRP78/mTOR axis in gastric cancer (185). Another study showed that autophagy develops a protective function in multi-drug resistant ovarian cancer cells mediated by vincristine, and the inhibition of autophagy resensitizes tumors cells to vincristine and restore its killing effects (186). Our group demonstrated that the overexpression of PTOV1, induced resistance in cells through autophagy activation, a fact appreciated in head and neck squamous carcinoma cell lines. Also, we observed that both in cell lines and head and neck cancer patients resistant to cisplatin, they overexpressed markers of autophagy and PTOV1. Of interest is that some of these markers had prognostic value when correlated with clinical variables (Figure 4). Besides, we suggested that the acquisition of resistance to cisplatin is related to the development of 5-fluorouracil resistance, supporting the presence of a common regulatory resistance pathway (187).

Moreover, it has been described that cisplatin or 5-fluorouracil promotes cytoprotective autophagy through upregulation of Beclin-1 in bladder cancer cells (188), an effect that has also been reported in other tumor cells, such as laryngeal, ovarian, esophageal, and colon cancer models (189–191). Upregulation of another autophagy-related gene, ATG7, also induces autophagy after treatment with cisplatin or 5-fluorouracil in esophageal cancer cells (192, 193). Of interest, the chemotherapeutic agents' cisplatin, temozolomide, and daunorubicin have been seen to stimulate protective autophagy by upregulation of the extracellular signal-regulated kinase (ERK) pathway, a mechanism observed in non-small cell lung cancer, ovarian cancer, glioma, and myeloid leukemia (194–198). Also, autophagy stimulation via AMPK, promoted by the chemotherapeutic agents' temozolomide, 5-fluorouracil, and docetaxel, confers resistance in different tumor types, such as prostate cancer, gastric cancer, and glioma (199–201). Besides, JNK upregulation induces autophagy-mediated chemoresistance to 5-fluorouracil in colorectal cancer cells (202).

**Figure 5:**
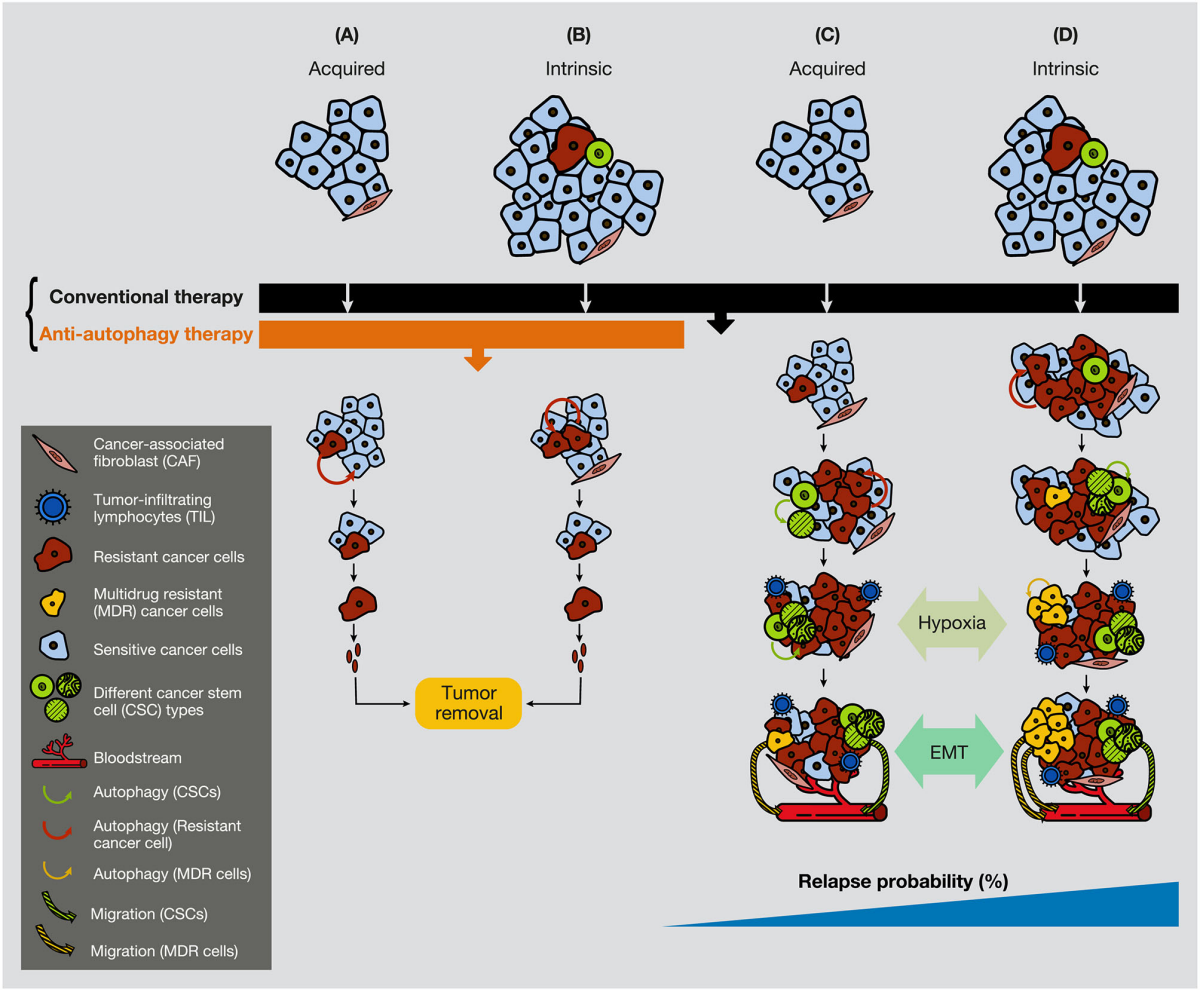
Anti-autophagic therapy can be efficient in the initial stages of tumors in the presence of resistance or CSCs. In the figure are depicted several scenarios where acquired or intrinsic resistance is already present in malignant tumors. **(A)** Acquired resistance appears consequently to the conventional treatment therapy, but it cannot progress due to the anti-autophagic therapy (e.g., hydroxychloroquine). **(B)** If resistant cells are present in the tumors, they cannot grow expansively because of the anti-autophagic therapy. **(C)** Acquired resistance appears consequently to the conventional chemotherapeutic treatment. Resistant cells survive largely supported by the activation of autophagy, either from surrounding cancer cells or from CAFs. **(D)** While conventional chemotherapeutic treatment annihilates the bulk of cancer cells, it favors the spread of resistant cells, including the CSCs. At a certain time of tumor development, the hypoxic conditions enable EMT process, allowing cancer cell plasticity to enter into the circulation. CAF, cancer-associated fibroblast; TIL, tumor-infiltrating lymphocyte; CC, cancer cell.

Furthermore, chemotherapeutic inhibition of the mTOR pathway induces an autophagy process that, in sensitive cells and after long-exposure treatment, provokes cell death. However, if cancer cells have already reached some degree of resistance, the inhibition of mTOR can also generate JNK activation, P-ERK upregulation, and Bcl-2/Bcl-xL phosphorylation in the breast, gastric and esophageal cancer, with subsequent activation of protective autophagy (203–205). Overall, while conventional chemotherapy treatment is used extensively against malignant tumors and is efficient against the bulk of cancer cells, a certain small population of cancer cells (resistant cells and CSCs) activates autophagy to survives therapy. Figure 5 illustrates that a small quantity of resistant cells or CSCs present in tumors can be annihilated if a combination of conventional therapy and anti-autophagic therapy is applied from the beginning of the treatment (Figure 5).

## Autophagy as a Target for Therapeutic Purposes: Inhibition or Stimulation?

**Figure 6:**
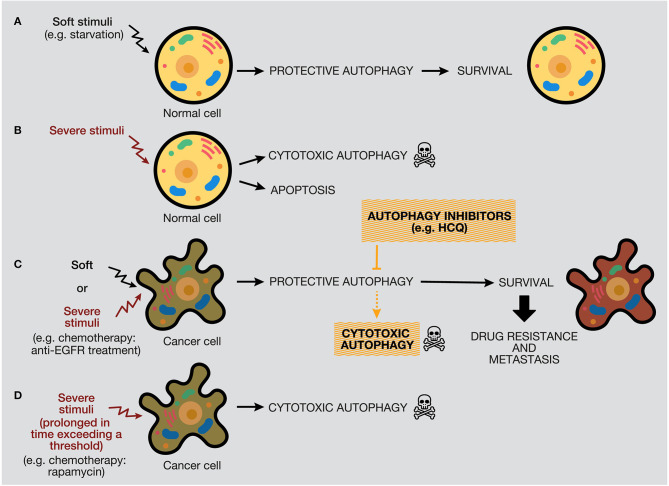
Distinctive responses of normal and cancer cells to different stimuli. The autophagy response depends on the intensity and duration of the stimuli (external or internal), highlighting a threshold in the autophagy mechanism that would determine cellular outcome. **(A)** Normal cells under soft stimuli, such as starvation, will ensure their survival through protective autophagy. **(B)** Severe stimuli on normal cells can induce cytotoxic autophagy or apoptosis. **(C)** In contrast, soft or severe stimuli over cancer cells (for example, anti-EGFR treatments) will provoke protective autophagy, which will confer survival properties, drug resistance, and metastasis. In this case, the use of autophagy inhibitors would provoke cell death by autophagy or occasionally by apoptosis. **(D)** Last, severe stimuli able to reach a threshold can increase cytotoxic autophagy.

The protective function of autophagy in healthy cells in response to soft or severe stimuli, such as starvation or hypoxia, acts as a protective mechanism to ensure cell survival, and if healthy cells cannot restore the damage, they will die by apoptosis (48). Thereby, autophagy activation acts as a tumor suppressor mechanism, preventing tumor initiation by maintaining metabolic homeostasis and suppressing genomic instability (Figures 6A,B). We propose a model where, if a severe stimulus occurs in cancer cells, such as chemotherapy or radiotherapy, protective autophagy emerges in most cases when apoptosis is defective (a common feature of cancer cells) (Figure 6C). Nevertheless, if the stress exceeds a threshold incompatible with cellular life, autophagy activation can mediate cell death depending on the genetic background, tumor evolution, and microenvironment (181). However, as mentioned above, the lack of consensus of whether there is a cytotoxic form of autophagy or stimulating autophagy in several tumor models under some circumstances just promotes or facilitates another type of cell death is a complex field (40, 206).

By one side, some anticancer treatments trigger autophagy as a death executioner, causing a cytotoxic effect that ends in autophagic cell death (30). This therapeutic strategy can be suitable in tumors with deficiencies in the activation of the apoptotic programmed cell death pathway, such as tumor cells lacking functional p53 (207). According to our model, a long-term autophagy activation strategy, generated by chemotherapy, radiotherapy, or targeted drugs, can be used to generate cytotoxic autophagy and cancer cell death (Figure 6D). For example, long-term treatment of PtAcacDMS, a novel platinum-based drug, triggers both apoptosis and autophagy, resulting in cell death in neuroblastoma cells (208). However, stimulation of autophagic cell death by different targeted drugs has been considered by researchers as an attractive alternative to treat some tumors that show some signs of apoptosis. The most representative example is the inhibition of the mTOR pathway as the main method to trigger autophagy in preclinical and clinical studies. For example, rapamycin is a selective inhibitor of mTORC1 and causes activation of autophagy (209). In several studies, rapamycin has been demonstrated to suppress cancer proliferation and induce autophagic cell death in different cancer models, such as neuroblastoma, osteosarcoma, and sarcoma (210–212). Rapamycin analogs (rapalogs), such as temsirolimus and everolimus, also inhibit the mTOR pathway in renal cancer and breast cancer, among others (213, 214). Other types of autophagy activators include the BH3 mimetics (e.g., gossypol, obatoclax), which provoke Bcl-2/Beclin-1 complex dissociation through interaction with Bcl-2 (215, 216). Histone deacetylase inhibitors, such as suberoylanilide hydroxamic acid (e.g., SAHA or Vorinostat), have also been implied in the activation of apoptosis and autophagy by inactivation of the PI3K/AKT/mTOR pathway (217, 218). Also, natural compounds have demonstrated antitumor properties through autophagy stimulation. For example, curcumin promotes autophagy-mediated cell death at high doses by the oxidative stress pathway. However, at low concentrations of curcumin, autophagy mediates cell protection through AMPK and ER stress pathways, evidencing a dual effect of curcumin, depending on the duration and concentration administered (219).

It is frequently observed that tumor cells activate autophagy to protect themselves from the stress caused by anticancer treatments, such as chemotherapy, radiotherapy, targeted therapy, or immunotherapy (220, 221). This activation occurs under prolonged treatments, due to the waiting time for patients to recover between different chemotherapy sessions; this process can also be considerably accelerated if the therapies are not effective. Therefore, upregulation of autophagy due to chemotherapeutic treatment in some cancer types (e.g., pancreatic cancer) or due to specific genetic conditions (e.g., Ras gene family mutations) promote drug resistance, which permits tumor recurrence, invasion, and metastatic development (3, 222). Our group has suggested that for particularly aggressive tumors, which might contain many mutations at the genetic and non-genetic levels, the activation of autophagy is a mechanism used by cancer cells to acquire an MDR phenotype (223). In this scenario, the inhibition of the autophagic process concomitantly to conventional therapy may be the appropriate strategy. The lysosomal inhibitors chloroquine (CQ) and its analog, HCQ, are the most extensive autophagy inhibitors in research and clinical studies (224). CQ and HCQ suppress autophagy via alteration of lysosomal pH and inactivation of acidic hydrolases, resulting in blocking of autophagolysosomal formation, accumulation of autophagosomes, and inactivation of autophagic degradation (157, 224, 225).

**Figure 7:**
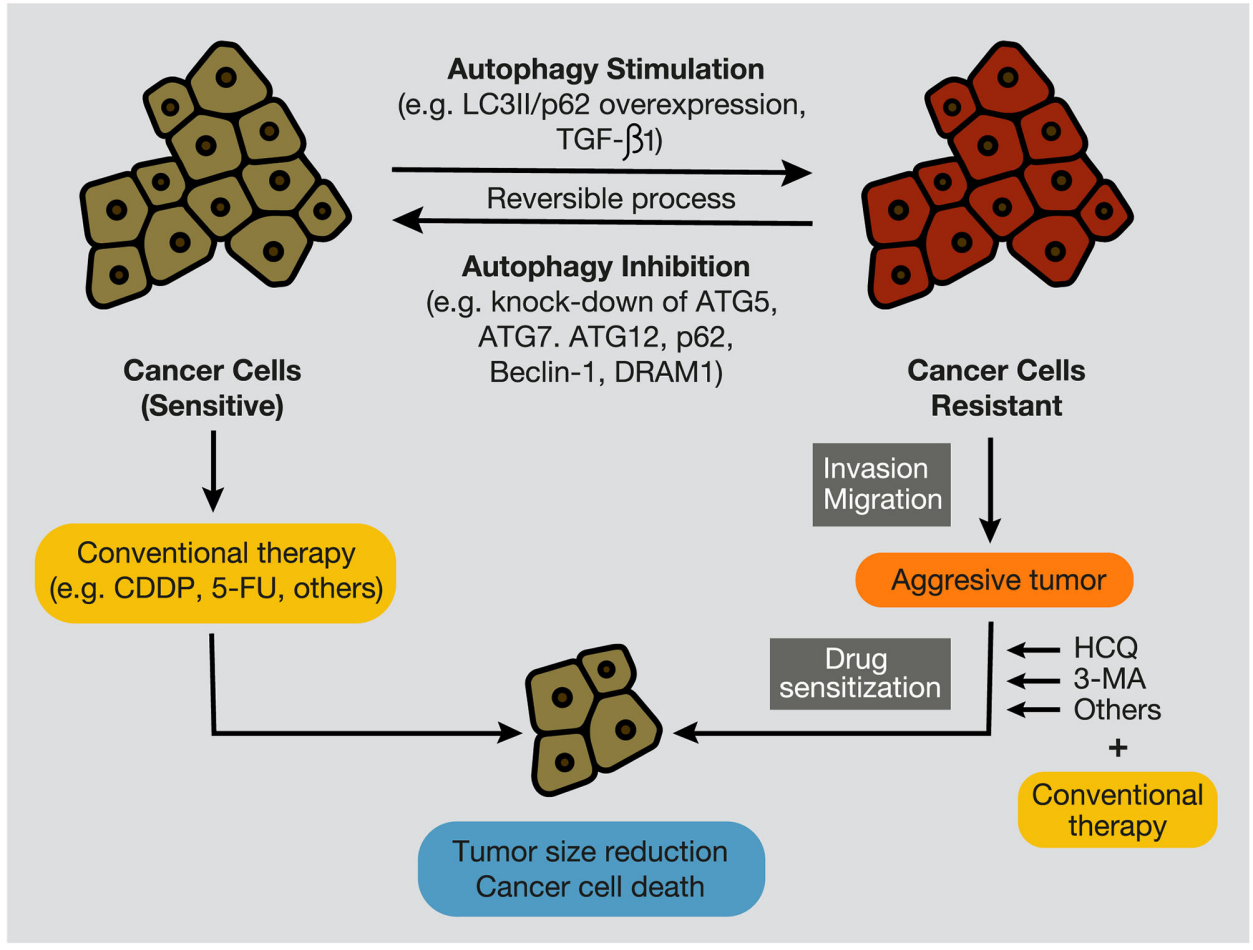
Autophagy stimulation and inhibition in cancer cells. During tumor development, autophagy inhibition, by targeting autophagy-related proteins, such as ATG7, Beclin-1, p62/SQSTM1, and DRAM1, promotes the sensitization of cancer cells to conventional anticancer treatments, such as chemotherapeutic agents, including CDDP and 5-FU. In contrast, autophagy stimulation, evidenced by overexpression of LC3II and p62/SQSTM1, and by high levels of TGF-β1, provokes cancer cell resistance to therapies (e.g., chemotherapy and radiation), development of an aggressive phenotype, and increment of migratory and invasive capacities. In this case, several autophagy inhibitors, such as CQ, HCQ, or 3-MA, can re-sensitize resistant tumors and promote tumor regression and cancer cell death. CQ, chloroquine; HCQ, hydroxychloroquine; 3-MA, 3-methyladenine; CDDP, cisplatin; 5-FU, 5- fluorouracil.

Moreover, the inhibition of the PI3KC3 complex is another strategy to inhibit autophagy in cancer cells (207). Wortmannin, 3-Methyl Adenine (3-MA), Spautin-1, and LY294002 have shown promising results in preclinical studies in coadministration with chemotherapeutics such as docetaxel, cisplatin, doxorubicin, or 5-fluorouracil (Figure 7). Resveratrol, which controls S6K1 and inhibits the ROS/ERK pathway, and 4-Acetylantroquinonol B, which reduces ATG-7 and ATG-5 expression, are two compounds used to reduce the autophagic process with promising preclinical outcomes. Moreover, HCQ treatment has been used to treat resistant cells to radiotherapy through *in silico*-designed nanoparticles for autophagy inhibition (226). Many studies in preclinical models (tumor cell lines and animal models) have demonstrated that CQ and HCQ induces cancer cell killing through treatment alone or in combination with targeted agents, radiotherapy, or chemotherapy (1). Besides, CQ and HCQ have been part, and are currently part, of several clinical trials in cotreatment with chemotherapeutics of different types of cancer, including glioblastoma, multiple myeloma, small and non-small cell lung, colorectal, pancreatic, prostate, and breast cancers (Table 1). Although clinical results to autophagy inhibition by CQ or HCQ has not been as consistent as seen in preclinical studies until now, the overall results published in clinical trials have proved their safe use as cancer therapy and their commitment to the biological target. Therefore, these autophagy inhibitors continue being used in active clinical trials in cotreatment with target therapy and chemotherapeutic drugs, including the International Cooperative Phase III Trial (HIT-HGG-2013) in Glioma and Gliomatosis Cerebri of temozolomide in cotreatment with valproic acid or CQ (NCT03243461).

**Table 1:** Anticancer drugs that inhibit autophagy used in combination with chemotherapy.

**Autophagy inhibitor**	**Function**	**Tumor type**	**Chemotherapeutic**	**(Pre-) clinical phase**	**NCT number**	**Ref**
Chloroquine	Inhibits acidification of lysosome and autophagosome-lysosome formation	Glioblastoma, Gliosarcoma	Temozolomide	I	NCT04397679	–
		Glioma, Gliomatosis Cerebri	Temozolomide	III	NCT03243461	–
		Glioblastoma, Astrocytoma	Temozolomide	II	NCT02432417	–
		Glioblastoma	Temozolomide	I	NCT02378532	–
		Solid Tumors	Carboplatin, Gemcitabine	I	NCT02071537	–
		Pancreatic Cancer	Gemcitabine	I	NCT01777477	(227)
		Multiple Myeloma	Cyclophosphamide	II	NCT01438177	–
		Glioblastoma	Not specified	III	NCT00224978	(228)
Hydroxy Chloroquine	Inhibits acidification of lysosome and autophagosome-lysosome formation	Osteosarcoma	Docetaxel, Gemcitabine	I/II	NCT03598595	–
		Pancreatic Cancer	Gemcitabine, Nab-Paclitaxel	II	NCT03344172	–
		Small Cell Lung Cancer	Gemcitabine, Carboplatin	II	NCT02722369	–
		Acute Myeloid Leukemia	Mitoxantrone, Etoposide	I	NCT02631252	–
		Pancreatic Cancer	Gemcitabine, Abraxane	II	NCT01978184	–
		Multiple Myeloma	Cyclophosphamide	I	NCT01689987	–
		Non-Small Cell Lung Cancer	Paclitaxel, Carboplatin	II	NCT01649947	–
		Pancreatic Cancer	Gemcitabine, Nab-Paclitaxel	I/II	NCT01506973	(229)
		Pancreatic Cancer	Capecitabine and Radiation	II	NCT01494155	–
		Colorectal Cancer	Oxaliplatin, 5-fluorouracil	I/II	NCT01206530	–
		Pancreatic Cancer	Gemcitabine	I/II	NCT01128296	(230)
		Colorectal Cancer	Capecitabine, Oxaliplatin	II	NCT01006369	–
		Prostate Cancer	Docetaxel	II	NCT00786682	–
		Breast Cancer	Ixabepilone	I/II	NCT00765765	–
		Non-Small Cell Lung Cancer	Carboplatin, Paclitaxel	I/II	NCT00728845	–
		Solid Tumors	Temozolomide	I	NCT00714181	–
		Glioblastoma	Temozolomide	I/II	NCT00486603	(231)
Wortmannin	Inhibits PI3KC3 complex and autophagosome formation.	Lung cancer, Prostate cancer	Docetaxel	Pre-clinical	NA	(232)
		Ovarian cancer	Cisplatin	Pre-clinical	NA	(233)
3-Methyl Adenine (3-MA)	Inhibits PI3KC3 complex and autophagosome formation.	Non-Small Cell Lung Cancer	Camptothecin	Pre-clinical	NA	(234)
		Colon cancer	Oxaliplatin	Pre-clinical	NA	(235)
Spautin-1	Enhances Beclin1 ubiquitination and prevent PI3KC3 complex formation	Melanoma	Cisplatin	Pre-clinical	NA	(236)
		Osteosarcoma cells	Doxorubicin	Pre-clinical	NA	(237)
LY294002	Inhibits PI3KC3 complex and autophagosome formation.	Oesophageal squamous cell carcinoma	5-fluorouracil	Pre-clinical	NA	(238)
Resveratrol	Regulates S6K1, inhibit ROS/ERK pathway	Glioma	Temozolomide	Pre-clinical	NA	(197)
		Ehrlich ascitic carcinoma	Doxorubicin	Pre-clinical	NA	(239)
4-Acetylantroquinonol B	Downregulation of ATG-7 and ATG-5	Ovarian cancer	Cisplatin	Pre-clinical	NA	(240)

*(–) information available at www.clinicaltrials.gov, NA: Not Apply*.

Overall, our model proposes that if acquired or intrinsic resistance is present at the initial stages of a tumor, it is possible to eradicate aggressive resistant cells by applying an autophagy inhibitory therapy from the beginning of the treatment concomitantly to conventional therapy. Personalized medicine to predict the status of autophagy (activated or defective) in cancer cells and the presence of specific markers able to predict the resistance or sensitization of cancer cells are key factors for predicting and choosing the best treatment for cancer patients. The incorporation of molecular (e.g., next-generation sequencing) and pathological (assessment of the overexpression of autophagy-related proteins or determination of the lymphocyte infiltration of tumors) techniques would improve the focus toward the most appropriate therapy.

## Conclusions

- The need to use, in general terms, high doses of conventional therapy to achieve therapeutic effects is the cause of the severe side effects of chemotherapies. As a result, chemotherapy sessions must be spaced to let patients recover from the side effects. This time-lapse is exploited by tumor cells to recover, proliferate, develop drug resistance, and create metastases responsible for most cancer deaths.
- How to tackle the acquisition of therapy resistance by tumors represents one of the most important challenges in cancer.
- Autophagy seems to favor cancer cells to acquire resistance; however, autophagy has a context-reliant function in cancer.
- Anti-autophagic treatments (e.g., HCQ) are very tolerable for patients and rarely cause severe side effects.
- It is of crucial importance that an effective treatment should be given to each cancer patient as the first therapeutic choice. Personalized medicine includes (a) the culturing of patient biopsies using spheroids, organoids, or mouse models to advance the benefits of a particular treatment and (b) the identification of genetic alterations by next-generation sequencing, which would point out specific drugs for particular mutations.

## Author Contributions

JA-M wrote the manuscript, YG-M, CM, and HK revised critically the manuscript, ML wrote the manuscript, revised it critically, and coordinated the work. All authors contributed to the article and approved the submitted version.

## Conflict of Interest

The authors declare that the research was conducted in the absence of any commercial or financial relationships that could be construed as a potential conflict of interest.
